# Association of Alzheimer’s Disease and Insulin Resistance in King Abdulaziz Medical City, Jeddah

**DOI:** 10.7759/cureus.19811

**Published:** 2021-11-22

**Authors:** Norah T Faqih, Albaraa F Ashoor, Sahl A Alshaikh, Alaa F Maglan, Nisreen Jastaniah

**Affiliations:** 1 Medicine, Umm Al-Qura University, Makkah, SAU; 2 Medicine, King Saud Bin Abdulaziz University for Health Sciences, Jeddah, SAU; 3 Geriatrics, King Abdulaziz Medical City, Ministry of National Guard-Health Affairs, Jeddah, SAU

**Keywords:** tyg index, dementia, metabolic syndrome, insulin resistance, alzheimer’s disease

## Abstract

Background

Alzheimer’s disease (AD) and insulin resistance (IR) are common in the elderly. IR reduces the ability of insulin to work effectively on target tissues. This results in hyperglycemia, increased triglyceride levels, decreased high-density lipoprotein (HDL) levels, elevated blood pressure, and central obesity, a condition known as metabolic syndrome (MetS). MetS eventually affects cognition, but its relationship with AD is unclear. Therefore, we studied the association between AD and IR and the relation between AD and diabetic patients treated with insulin.

Methods

This was a record-based retrospective cohort study using data from King Abdulaziz Medical City, Jeddah, Ministry of National Guards-Health Affairs. for all patients with dementia and AD, from 2009 to 2018. We examined 354 patient files. The triglyceride-glucose (TyG) index was used for the assessment of IR.

Results

There was no significant association between patients’ demographic data, glycated hemoglobin, and co-morbidities and developing AD. Statistical models showed that, after adjustment for age, patients with IR had a significantly higher likelihood of AD (adjusted OR = 1.4; 95% CI: 1.01-2.33). After multivariate adjustment, patients with IR still had a 20% higher probability of developing AD than others (adjusted OR = 1.2; 95% CI: 1.0-3.1).

Conclusion

These results suggest that AD is associated with IR. Moreover, the association may be confounded by many patient-related factors.

## Introduction

Alzheimer’s disease (AD) is a slowly progressing neurodegenerative disease [1]. It is characterized by mild memory loss that progressively leads to an inability to hold a conversation or respond to the environment [1]. At a microscopic level, there is a deposition of two protein types: senile plaques, an extracellular aggregation of beta-amyloid protein, and neurofibrillary tangles, an aggregation of tau protein in the intracellular compartment [1]. The accumulation of these proteins in the brain leads to neuronal death and brain atrophy, affecting areas like the hippocampus and the temporal and prefrontal cortex. This leads to the impairment of memory and cognition [1]. In the United States in 2021, the prevalence of AD among people aged 65-74 was 5.3%. This number increases significantly with age, affecting 34.6% of people aged 85 and older [2]. The prevalence of AD in the Arab world in 2019 was 1.1%-2.3% for people aged 50 years and above and 13.5%-18.5% for people aged 80 and above [3].

Insulin resistance (IR) essentially is the reduced ability of insulin to work effectively on target tissues such as muscle, liver, and fat. This hinders glucose uptake by insulin-sensitive tissues and increases hepatic glucose production, resulting in hyperglycemia [4]. According to the International Diabetic Federation (IDF) and American Heart Association/National Heart, Lung and Blood Institute (AHA/NHLBI), metabolic syndrome (MetS) is diagnosed when three or more of the following conditions exist: IR, raised fasting blood glucose, increased triglycerides, decreased high-density lipoprotein (HDL levels), elevated blood pressure, and central obesity [4-5]. However, IR is the main component of MetS [4]. The global prevalence of MetS is between 10% and 84%, depending on the region, ethnicity, age, sex, and race of the population. The IDF estimates that a quarter of the world’s population suffers from MetS [5]. According to a study by Al-Rubeaan et al., the prevalence of metabolic syndrome in Saudi Arabia was 39.8% and 31.6% based on the National Health and Nutrition Examination Survey (NHANES) ATP III criteria and IDF criteria, respectively [6].

Several hypotheses associate IR with AD via various pathological mechanisms. According to Akter et al. [7] and Jayaraj et al. [8]. Diabetes mellitus (DM) may be a cofactor in AD progression due to selective impairment in insulin production, metabolism, or signaling, accompanied by significant upregulation of tau hyperphosphorylation, β-amyloid aggregation, inflammation, mitochondrial dysfunction, and oxidative stress. Evidence for the association between IR and AD in Saudi Arabia is scarce, and there is an ongoing debate about the cost-effectiveness of clinical trials.

The triglyceride glucose (TyG) index is derived from fasting triglyceride levels and glucose as follows: ln [triglyceride (mg/dL) × fasting blood glucose (mg/dL)/2]. TyG has been suggested as a surrogate marker for use in the assessment of IR [9]. This study aims to determine the association between AD and IR in patients from King Abdulaziz Medical City, Jeddah (KAMC-J) and to study the relation between AD and diabetes mellitus treated with insulin.

## Materials and methods

Study design and patients’ records

This was a record-based retrospective cohort study conducted in KAMC-J, Ministry of National Guards-Health Affairs (NGHA). The study was conducted between March and July 2019. We identified 379 files of patients who were admitted to KAMC-J, with AD and dementia or memory loss during the period 2009 to 2018. We excluded 17 files that lacked complete data and eight files of young patients, patients with acute brain injury, or with cognitive impairment. In total, 354 patient files were analyzed in the present study.

Study database

Data for the analysis were obtained from the health information system database “BESTCare,” a system that depends on advanced software for all patients admitted to KAMC-J. Patient records contain sociodemographic information, historical details, laboratory and radiological data, management plans, and even mortality information. Additional information was collected from chart data files. Approval was obtained from the Institutional Review Board office of King Abdullah International Medical Research Center (KAIMRC) (IRBC/0258/20).

Definitions of the TyG index and Alzheimer’s disease

The TyG index was calculated based on the equation derived in previous studies as follows: ln [triglyceride (mg/ dL) × fasting blood glucose (mg/dL)/2] [9]. AD was defined according to clinical criteria for AD diagnosis, which includes the insidious onset and progressive impairment of memory and other cognitive functions concurrent with the prescription of anti-dementia medication. The anti-dementia medications included acetylcholinesterase inhibitors (rivastigmine, galantamine, or donepezil) or N-methyl-D-aspartate receptor antagonists (memantine), which are the most commonly used treatments for dementia.

Clinical and laboratory measurements

All patient files were reviewed using a pre-structured data sheet to avoid data extraction errors. The data retrieved included demographic data (age and gender), body mass index (BMI), calculated as body weight (kg) divided by the square of the body height (m^2^). Glucose levels (fasting and random blood glucose), glycated hemoglobin, diagnosis of DM, including type and date, diabetic medication, and the type of treatment, were also extracted. The laboratory results extracted included total cholesterol, triglycerides, HDL, and low-density lipoprotein cholesterol. Also extracted from the records were additional diagnoses such as renal disease, hypertension, ischemic diseases (either ischemic stroke or heart disease), and hypothyroidism. The diagnosis of AD, the date of the diagnosis, and the treatment received were recorded.

Statistical analyses

The extracted data were analyzed with the statistical software IBM SPSS version 22 (IBM Corp., Armonk, NY). All statistical analyses were done using two-tailed tests. A P-value less than 0.05 was taken as statistically significant. Descriptive analysis based on frequency and percent distribution was done for all variables, including the patient’s bio-demographic data, blood glucose profiles, and the prevalence of IR and AD. Crosstabulation was used to assess the distribution of Alzheimer’s disease among patients admitted to KAMC-J by their bio-demographic data. The crude odds ratio (OR) was calculated for each factor with a 95% confidence interval (95% CI). Multivariate and hierarchical logistic regression models were used to assess adjusted relations by calculating the adjusted odds ratio (ORA).

## Results

A total of 354 patient files were reviewed. The patients were aged from 37 to over 100 years with a mean age of 80.5 ± 10.2 years. There were 190 (53.7%) female patients. In all, 156 (44.1%) patients had a normal weight, 113 (31.9%) were overweight, and 85 (24%) were obese. Regarding co-morbidities, 194 (54.8%) were diabetic, 239 (67.5%) were hypertensive, 153 (43.2%) had ischemic heart disease, 57 (16.1%) had renal disease, and 53 (15%) had hypothyroidism (Table 1).

**Table 1:** Bio-demographic data of patients admitted to King Abdul-Aziz Medical City

Bio-demographic data	N	%
Age in years		
< 70	40	11.3%
70-79	111	31.4%
80-89	145	41.0%
90+	58	16.4%
Gender		
Male	164	46.3%
Female	190	53.7%
Body mass index		
Normal weight	156	44.1%
Overweight	113	31.9%
Obese	85	24.0%
Co-morbidities		
Diabetes mellitus	194	54.8%
Renal diseases	57	16.1%
Hypertension	239	67.5%
Ischemic heart disease	153	43.2%
Hypothyroidism	53	15.0%

All diabetic patients had type 2 DM. There were 35 (23%) patients on oral hypoglycemic medication while 87 (51.3%) were on insulin injections. A total of 121 (34.2%) patients had glycated hemoglobin exceeding 7% with a mean fasting blood glucose of 7.8 ± 3.9 and a random blood glucose level of 11.6 ± 8.4 (Table 2).

**Table 2:** Blood glucose profile among patients admitted to King Abdul-Aziz Medical City

Blood glucose profile	N	%
Diabetes mellitus type		
Type 2 diabetes mellitus	194	100.0%
Type of diabetic medication		
Oral hypoglycaemic	35	23.0%
Insulin	78	51.3%
Metformin	39	25.7%
Glycated haemoglobin		
< 7 (good control)	233	65.8%
> 7 (poor control)	121	34.2%
Fasting blood glucose		
Range	3.7-27.2	
Mean ± SD	7.8 ± 3.9	
Random blood glucose		
Range	2.8-89.3	
Mean ± SD	11.6 ± 8.4	

**Figure 1:**
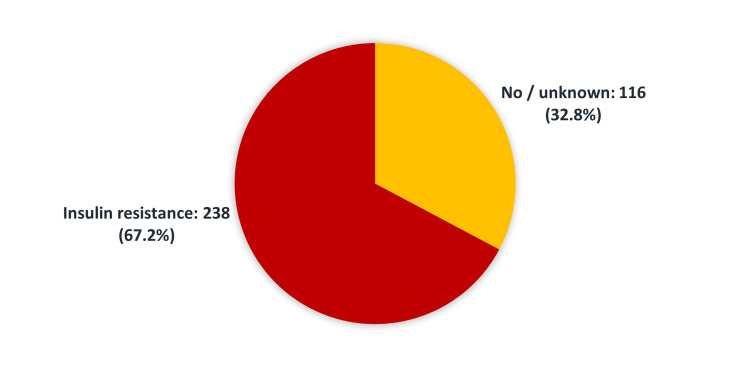
Prevalence of insulin resistance among patients admitted to King Abdul-Aziz Medical City

**Figure 2:**
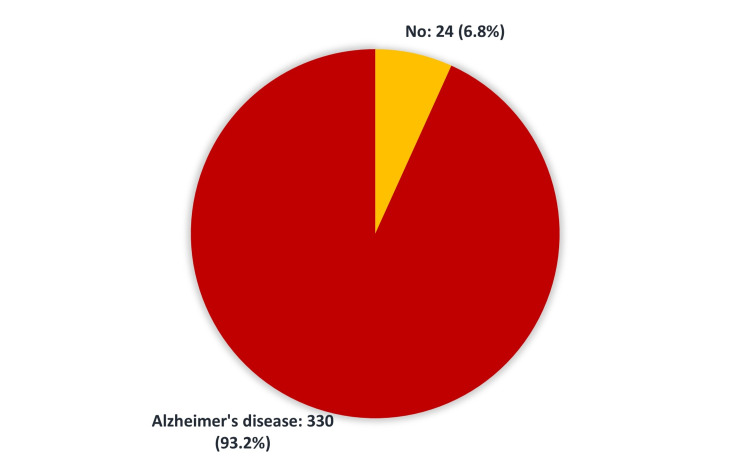
Prevalence of Alzheimer’s disease among patients admitted to King Abdul-Aziz Medical City

We identified 238 (67.2%) patients with insulin resistance (Figure 1), and 330 (93.2%) were diagnosed with Alzheimer’s disease (Figure 2).

AD was insignificantly more common among older patients (80-89 years) with an OR of 2.6, among females (OR = 1.4), and among obese patients (OR = 1.3). Although not reaching significance, patients with poor glycaemic control had a higher likelihood for AD (OR = 1.7), as did patients on insulin injection (OR=1.4) and patients with renal disease (OR=1.4) (Table 3).

**Table 3:** Distribution of Alzheimer’s disease among patients by their bio-demographic data OR=crude odds ratio, CI=confidence interval

Factors	Alzheimer’s disease				OR (95% CI)
	No		Yes		
	N	%	N	%	
Age in years					
< 70	4	10.0%	36	90.0%	1
70-79	10	9.0%	101	91.0%	1.2 (0.33-3.80)
80-89	6	4.1%	139	95.9%	2.6 (0.69-9.60)
90+	4	6.9%	54	93.1%	1.5 (0.35-6.38)
Gender					
Male	13	7.9%	151	92.1%	1
Female	11	5.8%	179	94.2%	1.4 (0.61-3.21)
Body mass index					
Normal weight	11	7.1%	145	92.9%	1
Overweight	8	7.1%	105	92.9%	0.99 (0.38-2.56)
Obese	5	5.9%	80	94.1%	1.3 (0.41-3.61)
Glycated haemoglobin					
≤ 7 (good control)	18	7.7%	215	92.3%	1
> 7 (poor control)	6	5.0%	115	95.0%	1.7 (0.62-4.15)
Diabetes mellitus					
No	9	5.6%	151	94.4%	1
Yes	15	7.7%	179	92.3%	0.71 (0.30-1.67)
Insulin injection					
No	20	7.2%	256	92.8%	1
Yes	4	5.1%	74	94.9%	1.4 (0.45-3.36)
Renal diseases					
No	21	7.1%	276	92.9%	1
Yes	3	5.3%	54	94.7%	1.4 (0.39-4.75)
Hypertension					
No	6	5.2%	109	94.8%	1
Yes	18	7.5%	221	92.5%	0.68 (0.26-1.75)
Ischemic heart disease					
No	12	6.0%	189	94.0%	1
Yes	12	7.8%	141	92.2%	0.75 (0.32-1.71)
Hypothyroidism					
No	20	6.6%	281	93.4%	1
Yes	4	7.5%	49	92.5%	0.81 (0.28-2.66)

In total, patients with IR were 30% more likely to develop AD than patients without, but no statistical significance was recorded (OR=1.3; 95% CI: 0.35-2.9). Statistical models showed that, after adjustment for age, patients with IR reported a significantly higher likelihood for AD than others (adjusted OR = 1.4; 95% CI: 1.01-2.33), and after adjusting for many patient’s demographic and clinical data, patients with IR still had 20% higher probability for developing AD than others (adjusted OR=1.2; 95% CI: 1.0-3.1) (Table 4).

**Table 4:** Association between Alzheimer’s disease patients and insulin resistance in King Abdulaziz Medical City (crude and adjusted) OR=crude odds ratio, CI=confidence interval; $=adjusted odds ratio for patients' ages; #=adjusted odds ratio for age, gender, body mass index, co-morbidities, insulin intake, glycated haemoglobin

Insulin resistance	Alzheimer’s disease				OR (95% CI)
	No		Yes		
	N	%	N	%	
No / unknown	9	7.8%	107	92.2%	1
Yes	15	6.3%	223	93.7%	1.3 (0.35-2.9)
^$^ Age adjusted risk (OR _A_; 95% CI)	1.4 (1.01-2.33) *				
^#^ Multivariate adjusted risk (OR _A_; 95% CI)	1.2 (0.99-3.1) *				

## Discussion

The current study was conducted to assess the association of IR with AD at KAMC-J and to study the relationship of AD with diabetes treated with insulin. There is increasing evidence to suggest that insulin irregularities and IR may play a significant role in AD pathophysiology and clinical symptoms [10-11]. Evidence suggests that insulin influences memory, a function mediated by the hippocampus and the adjoining medial temporal cortex [12-14]. Our results showed that more than two-thirds (67%) of patients had IR (Figure 1) while the vast majority (93.2%) were diagnosed with AD (Figure 2). AD is the most frequent form of dementia among older adults, characterized by significant cognitive and neuropathological defects [15-16]. In the present study, most Alzheimer’s patients were between the ages of 80 and 89 years (41.0%) (Table 1). Patients with AD are also more prone to changes in insulin metabolism than others. These patients may have an increased risk of IR and hyperinsulinemia [15-16]. Our analysis revealed no significant association between clinical and demographic data, including age, gender, BMI, glycated hemoglobin, insulin therapy, and co-morbidities, and having AD (Table 3).

Conversely, the adjusted analysis revealed a significant relationship between IR and AD, which ranged from 20% to 40% higher risk (Table 4). This means that the association between IR and AD may be confounded by several patient-related factors, including age and other clinical data (Table 4). The suggested associations have been studied by many researchers with conclusions consistent with the current study findings. We found that the AD frequency was higher in type 2 diabetic patients and obese individuals, and known mechanisms are emerging in these disorders [17-20]. Consistent with our present study, this showed most Alzheimer’s patients had been diagnosed with type 2 DM (n = 194) for many years (Table 2). Most of them had a good glycated hemoglobin level and were on insulin (Table 2) despite most having a normal BMI (n = 156) (Table 1).

In an animal study by Townsend et al. [21] and Bomfim et al. [22] involving intracerebroventricular injection of amyloid β oligomers (AβOs) in mice and monkeys, it was found that neural IR is mostly triggered by amyloid-β oligomers as shown in main cultures of hippocampal nerve cells. It is mediated by human tumor necrosis factor-alpha (TNF-α) activation and insulin receptor substrate inhibition with major impairment of synaptic plasticity, synaptic dysfunction, diminished synaptic plasticity, and synapse loss.

Many researchers have reported that the burden from DM (especially type 2 ), obesity, non-alcoholic steatohepatitis, and AD has increased over the past few years [23]. A higher risk of having cognitive impairment, dementia, or AD has been reported among patients with type 2 DM or obesity/dyslipidemia disorders [24-25]. AD is associated with progressive brain IR and insulin deficiency [26-27]. Using insulin sensitizer agents or intranasal insulin recovered cognitive performance in experimental models and human AD or cognitive impairment cases [24,26-27]. Some molecular, biochemical, and mechanical abnormalities in type 2 DM and AD are shared [28]. Antonio et al. discovered in a case-control study that patients with AD have a seven-fold probability, compared to those who do not have AD, of having abnormalities in their glucose metabolism (72.2% vs. 37.8%, respectively) [29]. This is consistent with the present study that revealed fasting and random blood glucose abnormalities in all patients with AD (Table 2). Also, the Antonio et al. study found hypertriglyceridemia (61.1% vs. 48.9%) and low HDL cholesterol (46.7% vs. 34.4%) in AD and non-demented patients, respectively. However, they found that abnormalities in glucose metabolism were the only component of metabolic syndrome statistically associated with AD [29].

Little research has been done on this specific topic of IR. And because of our limited resources and the availability of specific parameters (two laboratory results) in the patient records, we were able to use a simple and validated tool, the TyG index. This is a compound index that combines fasting blood glucose (FPG) and fasting triglyceride (TG) for the assessment of IR [9]. There are several methods for IR estimation. The hyperinsulinemic-euglycemic clamp technique is considered the gold standard for research purposes [30]. However, it is invasive and requires intravenous injection of insulin and glucose in addition to continuous blood collection over multiple hours [30].

For this reason, there are other simple and validated methods, such as the McAuley, Belfiore, Cederholm, Avignon, and Stumvoll indexes, which are suitable for epidemiological research [30]. The Homeostasis Model Assessment, the Quantitative Insulin Sensitivity Check Index, and Matsuda are suitable for clinical use [30]. However, we could not use these in our target Alzheimer patients because the study design was retrospective. Further, the parameters for each of these indices are not commonly tested for in Alzheimer patients, as the cause of admission would not usually require it. Several studies that have been done to assess IR have used the TyG index, which is widely accepted and used clinically [9,30]. Indices of IR are increasing, and it may be difficult for researchers to select the most appropriate one for their studies. We encourage researchers to compare these indices for IR estimation for accuracy and sensitivity in the estimation of IR.

Limitations

The present study had several limitations. First, the small sample size may not represent the entire population in Jeddah city. Second, the retrospective study design had inherent limitations, although the multivariate analyses adjusted for some of the factors that could have affected the results. Third, IR was not directly measured. Although previous studies have used the TyG index for the assessment of IR with significant results, some inaccuracies may exist.

## Conclusions

Our record-based retrospective cohort study over an average of 10 years of 354 patient files showed a higher TyG index associated with AD. The TyG index is a surrogate marker of IR. The pathophysiological mechanisms that explain this association remain unclear. The study also showed that patients with IR had a higher probability of developing Alzheimer’s disease after adjustment for all confounders such as age and demographic and clinical data. This suggests that the association between IR and AD may be confounded by several patient-related factors. Moreover, there was no significant association between developing Alzheimer’s disease in diabetic patients on insulin injections.
